# Exercise tolerance through severe and extreme intensity domains

**DOI:** 10.14814/phy2.14014

**Published:** 2019-03-01

**Authors:** Andrew M. Alexander, Kaylin D. Didier, Shane M. Hammer, Alex C. Dzewaltowski, Karly N. Kriss, Garrett M. Lovoy, Joseph L. Hammer, Joshua R. Smith, Carl J. Ade, Ryan M. Broxterman, Thomas J. Barstow

**Affiliations:** ^1^ Department of Kinesiology Kansas State University Manhattan Kansas

**Keywords:** Central fatigue, critical power, extreme exercise, peripheral fatigue, severe exercise

## Abstract

The power‐duration relationship accurately predicts exercise tolerance for constant power exercise performed in the severe intensity domain. However, the accuracy of the prediction of time to task failure (*T*_lim_) is currently unclear for work rates (WR) above severe intensities; that is, within the extreme intensity domain (*T*_lim_ < 2 min). We hypothesized that *T*_lim_ would be shorter for WRs within the extreme intensity domain than predicted from the linear 1/time relationship of the severe intensity domain which would suggest mechanisms limiting exercise are different between intensity domains. Six men completed 7 knee‐extension tests. *T*_lim_ of extreme intensity exercise (60%, 70%, 80%, and 90% 1RM;* T*_lim_ < 2 min) were compared to the predicted *T*_lim_ from the slope of the S1–S3 (*T*_lim_ ≥ 2–15 min) regression. Twitch force (*Q*_tw_) and maximal voluntary contraction (MVC) were measured on the right vastus lateralis before and after each test. *T*_lim_ at 70–90% 1RM were shorter than the *T*_lim_ predicted by the severe domain 1/time model (*P* < 0.05); however, *T*_lim_ at 60% 1RM was not different than the predicted severe *T*_lim_, suggesting the mechanisms limiting extreme exercise manifest ≥60% 1RM. A significant linear relationship for 60–90% 1RM was observed which suggested a curvature constant unique to the extreme domain ($W_{\mathrm{ext}}^{\prime }$) that was smaller than the *W* ′ of the severe domain (1.5 ± 0.6 vs. 5.9 ± 1.5 kJ,* P* < 0.001). *Q*_tw_ and MVC were significantly decreased following severe exercise, however, *Q*_tw_ and MVC were not significantly decreased following 80% and 90% 1RM, giving evidence that mechanisms causing task failure were recovered by the time post‐exercise measurements were made (~90 sec).

## Introduction

Within the severe‐intensity domain, the power‐duration relationship is hyperbolic, with task failure occurring much sooner at higher compared to lower intensities (Tornvall 1963; Moritani et al. 1981; Poole et al. 1988). The asymptote of this relationship has been termed critical power (CP), and represents the lower limit of the severe intensity domain (Tornvall 1963; Monod and Scherrer 1965; Moritani et al. 1981; Vanhatalo et al. 2010; Broxterman et al. 2014). The curvature constant of the power‐duration relationship is a derivative of work, and has therefore been termed *W*′, and represents a finite work capacity above CP (Moritani et al. 1981; Poole et al. 1988). Due to the hyperbolic nature, this relationship can be expressed using a linear model by plotting power as a function of exercise tolerance (*T*_lim_) as 1/*T*_lim_, where the *Y*‐intercept is CP and the slope is *W*′. Importantly, given the parameters CP and *W*′, exercise tolerance (as time to task failure; *T*_lim_) in the severe domain is highly predictable.

Historically, 3–4 bouts of exercise eliciting *T*_lim_ ranging from 2 to 20 min have been used to determine CP and therefore the lower boundary of the severe intensity domain (Poole et al. 1988; Fukuba et al. 2003; Broxterman et al. 2014). Hill et al. (2002) proposed the need for an additional, supra‐severe exercise domain, where exercise intensity was so great that *T*_lim_ would be shorter than predicted by the severe intensity power‐duration relationship. This domain was dubbed the “extreme” domain (Hill et al. 2002), and it was predicted that *T*_lim_ would typically be reached in less than ~2 min. Furthermore, it may be possible that evidence of this separate domain is seen in other exercise modalities (Reynolds et al. 2006; Desgorces et al. 2010). Previously, exhaustive exercises with high resistance, low repetitions (~75–85% 1RM) have been shown to better predict one repetition maximum (1RM) than low resistance, high repetition (~20–60% 1RM) exercises for untrained and athletic populations (Reynolds et al. 2006; Desgorces et al. 2010). This suggests that the factors that contribute to task failure may be different above 60% 1RM compared to intensities below 60% 1RM and, importantly, *T*_lim_ for exercise ≥60% 1RM would occur in less than 2 min (Reynolds et al. 2006; Desgorces et al. 2010). Therefore, the deviation from the severe domain power‐duration relationship may occur at ~60% 1RM.

The relationship between exercise intensity and *T*_lim_ has been extensively studied in an attempt to identify the mechanisms of exercise intolerance (Bigland‐Ritchie et al. 1978, 1986; Stackhouse et al. 2000; Chidnok et al. 2012; Broxterman et al. 2015a, 2017a). In order to estimate the location of the fatigue during exercise, researchers have investigated central (i.e., proximal to the neuromuscular junction) and peripheral (i.e., at or distal to the neuromuscular junction) fatigue (Bigland‐Ritchie et al. 1978, 1986; Kent‐Braun 1999; Burnley 2009) using electrical stimulation. Following sustained maximal contractions, central fatigue, as measured by percent of voluntary activation (%VA), may only account for up to 30% of force decline (Bigland‐Ritchie et al. 1978; Kent‐Braun 1999), suggesting intramuscular milieu (i.e., peripheral fatigue) is largely responsible for the decline in force production during repeated maximal voluntary contractions (MVC). Furthermore, Bigland‐Ritchie et al. (1986) found the decline of MVC force following isometric voluntary contractions at 50% MVC to task failure was primarily caused by peripheral fatigue, as seen by the decrease in potentiated twitch force (*Q*_tw_) with little to no central fatigue present (Bigland‐Ritchie et al. 1986), consistent with the work of Yoon et al. (2007) at 80% MVC. However, it is currently unclear if the mechanisms (i.e., peripheral and central fatigue) limit exercise tolerance to a similar degree between severe and extreme exercise but with a lower threshold for extreme exercise.

Therefore, the first aim of the current study was to examine the power‐duration relationships of the severe and extreme intensity domains. The second aim was to quantify the relative contributions of peripheral and central fatigue throughout these intensity domains. We hypothesized that during dynamic knee extension exercise (1) *T*_lim_ for a given force or power in the extreme intensity domain would be shorter than predicted by the severe intensity power‐duration relationship beginning around 60% 1RM, (2) throughout the severe and extreme domains, *Q*_tw_ and MVC would be significantly decreased to a similar degree for all exercise intensities, whereas (3) there would be little to no change in %VA at task failure in either domain.

## Methods

### Subjects

Six healthy men (mean ± SD: 22.0 ± 3.1 year; 72.5 ± 6.5 kg; 178 ± 2 cm) participated in this study. All participants were free from cardiovascular, pulmonary, and metabolic disease as determined by a medical history questionnaire. Prior to participation in this study, subjects were informed of all procedures, and associated potential risks and benefits. Written informed consent was obtained from all participants prior to participation. Subjects were instructed to refrain from vigorous exercise 24 h, alcohol consumption 12 h, and food and caffeine 2 h prior to each session. Subjects were instructed to maintain current exercise habits in order to avoid any training or detraining effect. All research components were reviewed and approved by the Institutional Review Board of Human Subjects at Kansas State University, Manhattan, KS.

### Experimental design

Subjects visited the laboratory a minimum of 9 times, with at least 48 h between sessions. Subjects were first familiarized with all testing procedures and equipment prior to testing. All exercise tests were performed on an iso‐lateral knee extension machine (MTSLE Iso‐Lateral Leg Extension Machine, LifeFitness, Rosemont, IL) that was customized to perform simultaneous bilateral knee extension exercise. The subjects were seated on the knee extension machine with a hip flexion angle of 90° and a knee flexion angle of 80°. Seat position was replicated for each subject and exercise session.

### Determination of 1RM

Following familiarization, the first session consisted of a one repetition maximum (1RM) test, defined as the heaviest weight lifted through the entire pre‐determined range of motion (75°). The subjects began with a warm‐up of 8–10 repetitions at 9.09 kg. Initial weight was selected following feedback from the participant pertaining to their exercise history. Each subsequent increment was selected based on feedback from the subject. The minimum increase in weight was 4.54 kg due to the design of the exercise machine. No more than 2 attempts were allowed at a single resistance. A minimum of 5 min of rest was required between each attempt (Mayhew et al. 1995; Reynolds et al. 2006).

### Determination of peak power

In order to make comparisons to peak incremental power (*P*_peak_), the subjects performed a conventional incremental resistance test (Broxterman et al. 2014) to task failure to determine *P*_peak_ during the second visit to the laboratory. Subjects performed knee extension exercise at a 50% duty cycle (1.5 sec contraction; 1.5 sec relaxation) at a rate of 20 contractions/min as per a pre‐recorded audio cue. Initial resistance was set to 9.09 kg and was increased by 4.54 kg every minute until task failure. Subjects paused the contraction cycle during the last 5 sec of each minute to allow resistance to be increased. Task failure was determined when the subject failed to maintain contraction pace or complete the full range of motion for 3 consecutive contractions.

### Constant‐load tests

During each subsequent visit, subjects performed constant‐load tests to *T*_lim_ at 60, 70, 80, and 90% 1RM on separate visits in randomized order. Following the tests on subsequent days, subjects performed a minimum of 3 constant‐load exercise tests of increasing resistance (S1–S3; respectively) at intensities predicted to elicit a *T*_lim_ of 2–15 min, on separate days in randomized order. If exercise continued for >20 min, the exercise intensity was considered to be below CP, the test was terminated, and the subject returned after 48 h to perform a test at a higher work rate. A 1RM using the resistance found during the first session was performed 5 min prior to each exercise bout to normalize EMG signal. Subjects were allowed to set their own contraction frequency. However, relaxation between contractions was fixed at 1 sec to standardize time for blood flow (Broxterman et al. 2014).

*T*_lim_ of S1–S3 was used to calculate CP via the power‐duration relationship. Power throughout each constant‐load test was found using the equation:

P=(W∗d)/Contraction time

where “*W*” is the product of the mass of the resistance (kg) and the gravity constant (i.e., 9.8 m/sec^2^), “*d*” is the fixed distance that the weight moved (0.345 m), and “Contraction Time” is the total time from beginning of one contraction to the beginning of the next consecutive contraction.

### Neuromuscular function

Neuromuscular function testing was conducted similar to previous protocols used in our laboratory (Broxterman et al. 2015b). Briefly, testing was performed on the right leg prior to and following each constant load exercise test. The right ankle was secured to a force transducer (LBG1, BLH Electronics, Waltham, MA). Ankle height was adjusted for each subject such that a 90° angle of pull was maintained. The height was recorded and replicated for all future sessions. Adhesive electrodes (4 × 6 cm) were used to electrically stimulate the right quadriceps muscle via the femoral nerve. The anode was attached to the gluteal fold and the cathode was positioned over the approximate location of the femoral nerve (Babault et al. 2001), located by palpation of the femoral artery proximal to the femoral artery bifurcation. Before beginning each exercise protocol, the placement of the cathode that produced the greatest force development with electrical stimulation was determined and used for pre‐ and post‐exercise testing. Force was sampled at 1000 Hz and displayed on a computer screen (LabVIEW, National Instruments, Austin, TX). The quadriceps muscle was stimulated using a high‐voltage constant‐current electrical stimulator (DS7AH, Digitimer, Welwyn Garden City, UK). Paired stimuli (doublets) were delivered at 400 V with 100 *μ*sec square‐wave pulse durations and a 10 msec pulse interval. Maximal stimulation was assessed prior to each exercise bout. Stimulation intensity was initiated at 50 mA and was increased in 25 mA increments until the measured force and compound muscle action potential (M‐wave) ceased to increase. The stimulator current was then increased an additional 30% to ensure the stimuli were supramaximal. Prior to each exercise test, subjects performed a series of six, 3 sec maximal voluntary contractions (MVCs), beginning every 30 sec. Doublet muscle stimulations were delivered 5 sec prior to each MVC, 1.5 sec into the MVC, and 5 sec after each MVC to obtain measurements of unpotentiated, superimposed, and potentiated doublet forces, respectively. MVC was determined as the greatest force attained prior to the superimposed muscle doublet stimulation. This neuromuscular assessment was completed a second time starting at 30 sec following task failure in all but one test (80 sec following task failure). Thirty seconds represented the minimum amount of time required to transfer the subject from the knee extension ergometer to the force transducer in order to make the measurements. Due to previous data suggesting the degree of potentiation is lessened after the first two MVCs, the last four sets were used for data analysis (Kufel et al. 2002; Broxterman et al. 2015b).

### Electromyography

Surface EMG measurements were obtained during each session using a commercially available system (Trigno EMG, Delsys Inc., Boston, MA). Each EMG sensor contained four electrodes (5 × 1 mm) arranged in a 2 × 2 orientation to make single differential measurements. The belly of the right vastus lateralis was identified and placement of the sensor was marked with indelible ink to ensure repeatability of placement. The sensor was secured using an adhesive film. The EMG data were collected at a sampling rate of 1000 Hz and band‐pass filtered (13–400 Hz) using a fifth‐order Butterworth filter. The EMG signal corresponding to each muscle contraction was detected using previously developed (in house) software (MATLAB R2011a, The Mathworks, Natick, MA). The amplitude characteristics were described using the root mean squared (RMS) to provide an index of muscle activation and motorneuron firing rate. The frequency characteristics were described via median power frequency (MedPF) to provide an index of the muscle action potential conduction velocity. The EMG data were analyzed using binned averages of 3 contractions.

### Statistical analysis

**Figure 1:**
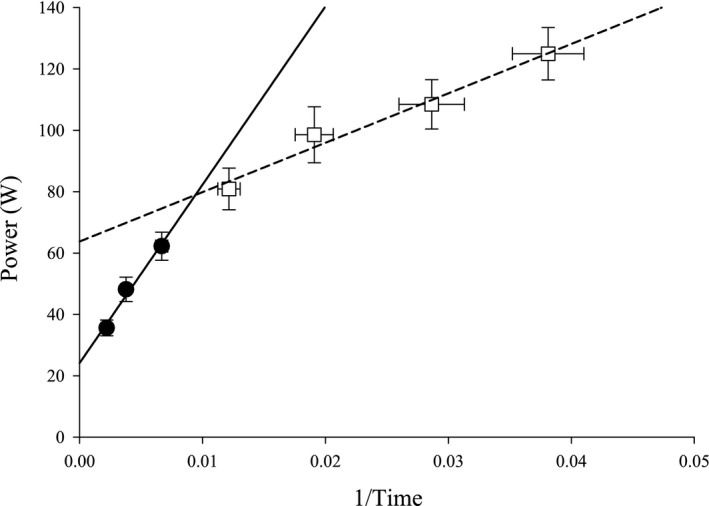
Average severe and extreme power‐duration relationships. Severe and extreme intensity power‐duration relationships for each subject shown by linear transformation. S1–S3 (●) regression shown by solid line. 60–90% 1RM (□) regression shown by dashed line. T_lim_ of 60% 1RM was not significantly different from the Tlim predicted by the S1–S3 regression (*P* = 0.39), but T_lim_ of 70–90% 1RM were significantly shorter.

**Figure 2:**
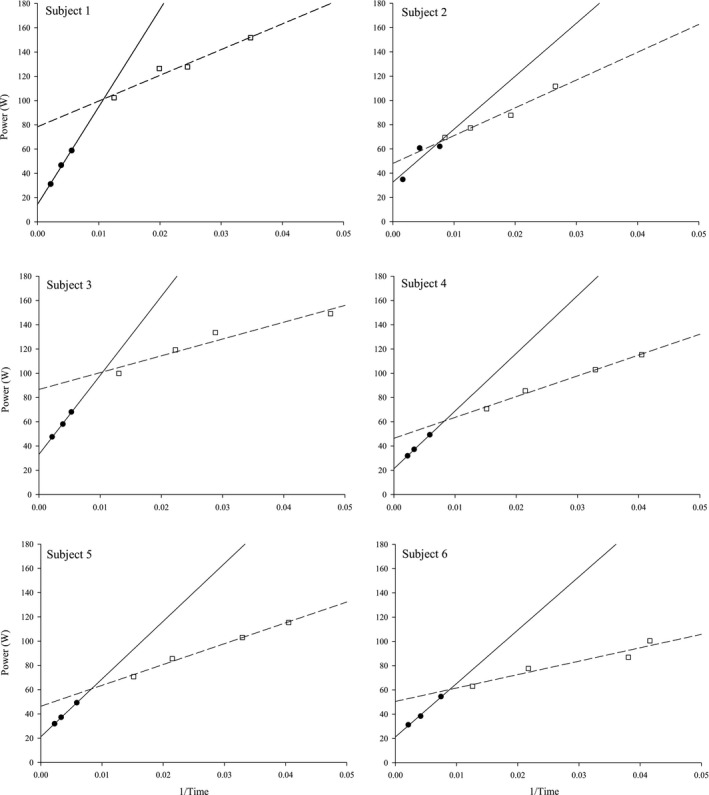
Individual severe and extreme power‐duration relationships. Severe and extreme intensity power‐duration relationships shown by linear transformation. S1– S3 (●) regression shown by solid line. 60–90% 1RM (□) regression shown by dashed line.

Based on visual inspection of the data in Figures 1 and 2, linear regression was applied to two regions of the power versus 1/*T*_lim_ responses for each subject: S1–S3 to determine the severe intensity relationship, and a second regression for the 60–90% 1RM work rates to determine the extreme intensity relationship. Actual *T*_lim_ for 60–90% 1RM were then compared to the *T*_lim_ predicted by the severe intensity regression using a two‐way ANOVA with repeated measures (intensity, actual vs. predicted). A two‐way ANOVA with repeated measures was used to test for differences in duration of contraction using EMG burst length time across all work rates and between the average of the first 5 contractions compared to the average of the last 5 contractions (intensity, first vs. last). A two‐way ANOVA was used to test for day‐to‐day differences in the pre‐exercise value of the MVC (intensity, day) for potential training adaptations. A one‐way ANOVA with repeated measures was used to test the change from baseline among intensities for *Q*_tw_, MVC, and %VA. Differences were considered statistically significant when *P* < 0.05. Data were reported as means ± standard deviation (SD) unless otherwise noted.

## Results

Bilateral knee extension 1RM was 108 ± 21 kg. Isometric, single leg knee extension MVC force measured during the pre‐exercise neuromuscular assessments was 44 ± 16% 1RM. There were no significant day‐to‐day differences in MVC (*P* = 0.13). The mean pre‐exercise coefficient of variation for MVC for all 7 exercise tests was 11%, with a range of 7–19%. Mean ± SD resistance and power for each intensity are shown in Table 1. S1 was 26 ± 3% 1RM, S2 was 34 ± 4% 1RM, and S3 was 44 ± 7% 1RM. P_peak_ (51 ± 7 W) was at 46 ± 6 kg or 43 ± 5% 1RM. The average EMG burst time of the first 5 contractions was not different from the average EMG burst time of the last 5 contractions for any subject (*P* = 0.46), and was independent of intensity (*P* = 0.38). Since relaxation time was fixed at 1 sec, overall contraction time, and thus duty cycle, was constant across work rates within each subject, that is, there was a constant relationship between force and power. We chose to present the data as power.

**Table 1:** Times to task failure

	Resistance (kg)	Power (W)	*T*_lim_ (s)
	Mean ± SD	Mean ± SD	Mean ± SD
1RM	108 ± 21		
90% 1RM	98 ± 19	125 ± 21	27 ± 6
80% 1RM	87 ± 16	108 ± 20	37 ± 9
70% 1RM	76 ± 15	98 ± 21	55 ± 13
60% 1RM	64 ± 13	81 ± 17	85 ± 18
S3	48 ± 9	62 ± 11	154 ± 29
S2	36 ± 6	48 ± 20	269 ± 38
S1	28 ± 5	36 ± 6	468 ± 86

Mean ± SD resistance, power, and time to task failure (*T*_lim)_ for all intensities. S1–S3 = severe intensity exercises (*T*_lim_ > 2 min).

### *T*_lim_ for severe and extreme domains

Mean ± SD *T*_lim_ for each intensity is shown in Figure 1, while individual data are shown in Figure 2. CP was 29 ± 7 W (19 ± 4% 1RM; 43 ± 7% *P*_peak_), while *W*′ was 5.9 ± 1.5 kJ. Post hoc analysis of the extreme domain (60–90% 1RM) revealed a significant linear relationship between power and 1/*T*_lim_ (*r* ^2^ = 0.94 ± 0.03). Using the interpretation that a hyperbolic relationship in the severe domain yields a derivative of work, these data show a *W*’ of the extreme domain ($W_{\mathrm{ext}}^{\prime }$) of 1.7 ± 0.4 kJ, which is significantly less than *W*′ for the severe domain (*P* < 0.003). *T*_lim_ for 70–90% 1RM were all significantly shorter (*P* < 0.05) than predicted by the severe domain 1/Time model, while the *T*_lim_ for 60% 1RM was not different (*P* > 0.05). The intersection of the severe (S1–S3) and extreme (60–90% 1RM) regression lines occurred at 79 ± 19 W (not significantly different from the value for 60% 1RM, *P* > 0.05) and *T*_lim_ was on average 112 ± 14 sec.

### Neuromuscular function

**Figure 3:**
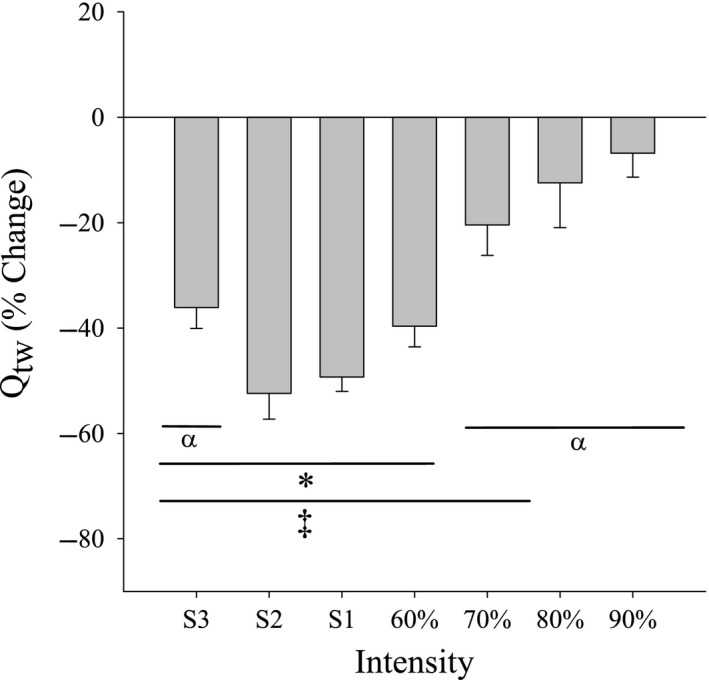
Twitch force pre‐ to post‐exercise. Percent change in potentiated twitch (*Q*_tw_) post‐exercise for all intensities (Mean ± SE). *Q*_tw_ following exercise at 80% and 90% did not significantly decrease (*P* = 0.34). ‡Different from zero (*P* < 0.01); *different from 70%, 80%, and 90% 1RM (*P* < 0.01); *α* different from S2 (*P* < 0.01).

The post‐exercise changes in potentiated twitch force (*Q*_tw_) are shown in Figure 3 for each intensity. Compared to resting baseline, the *Q*_tw_ was significantly reduced following the three severe exercise intensity tests (S1–S3), 60% 1RM and 70% 1RM (all *P* < 0.01). However, there was no significant decline from baseline in *Q*_tw_ following exercise at 80% or 90% 1RM (*P* = 0.09, *P* = 0.34; respectively), suggesting that if peripheral factors limited exercise at these intensities, the muscle was able to fully recover by the time the reported post‐exercise measurements were made (~90 sec). (N.B. We may have been underpowered, at least at 80% 1RM, to see a significant difference.)

**Figure 4:**
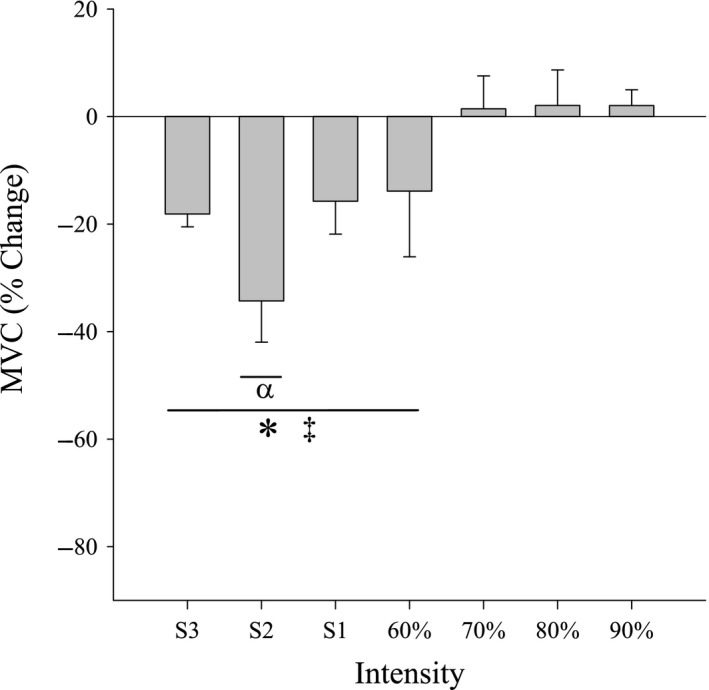
Maximal voluntary contraction pre‐ to post‐ exercise. Percent change in maximal voluntary contraction (MVC) post‐exercise for all intensities (Mean ± SE). MVC following exercise at 70%, 80%, and 90% did not significantly decrease (*P* = 0.76). ‡Different from zero (*P* < 0.05); *different from 70%, 80%, and 90% 1RM (*P* < 0.05); *α* different from all other intensities (*P* < 0.05).

**Figure 5:**
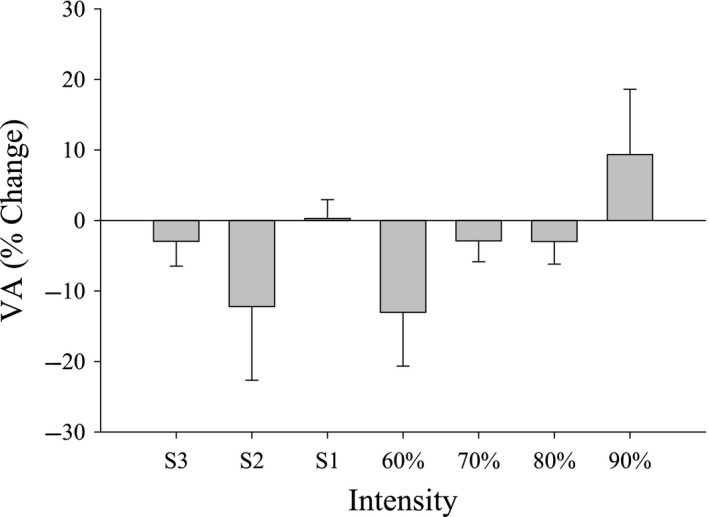
Voluntary activation pre‐ to post‐exercise. Percent change in voluntary activation (VA) post‐exercise for all intensities (Mean ± SE). No differences were detected in the percent change pre‐ to post‐exercise or the percent change among intensities.

Changes in MVC post‐exercise for each intensity are shown in Figure 4. MVC was significantly reduced for S1–S3 and 60% 1RM compared to baseline (*P* < 0.05). However, MVC was not significantly reduced following exercise at 70, 80, or 90% 1RM (*P* = 0.76). Figure 5 illustrates post‐exercise values of %VA for each intensity. There were no significant changes pre‐ (90.9 ± 3.9%) to post‐exercise (87.9 ± 9.2%) across intensities in %VA.

**Figure 6:**
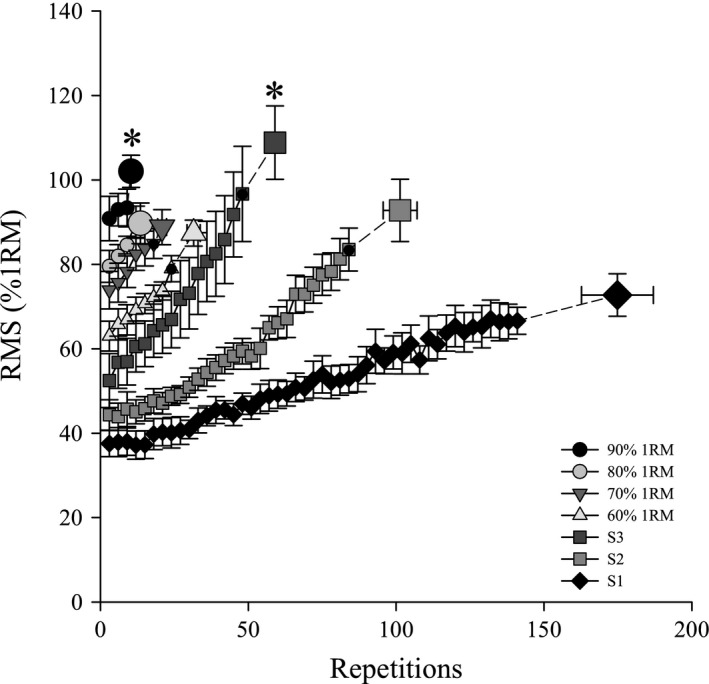
Root mean square throughout exercise. Changes in root mean square (RMS) as a percent of 1RM throughout each intensity and at end exercise. Data were averaged into 3 contraction bins. RMS increased throughout each intensity so that end exercise was significantly greater than at the beginning of exercise (*P* < 0.05). *Significantly greater than S1.

**Figure 7:**
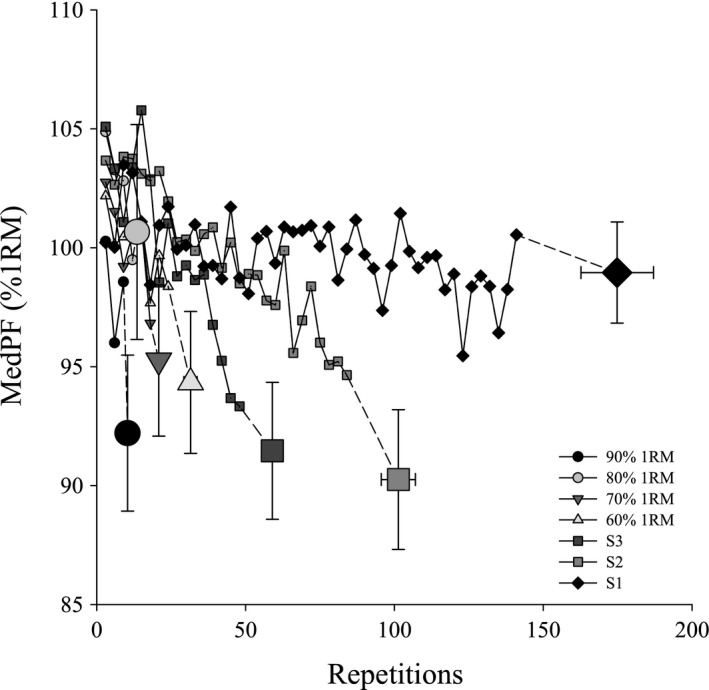
Median power frequency throughout exercise. Changes in median power frequency (MedPF) as a percent of 1RM throughout each intensity and at end exercise. Data were averaged into 3 contraction bins. MedPF decreased throughout each intensity so that end exercise was significantly less than at the beginning of exercise (*P* < 0.05). There were no differences among tests at end exercise.

Root mean square (RMS) and Median power frequency (MedPF) responses during each exercise test are shown in Figures 6 and 7, respectively. RMS significantly increased and MedPF significantly decreased throughout each intensity. End exercise RMS following 90% 1RM and S3 were significantly greater than for end exercise S1. End exercise MedPF was not different among tests.

## Discussion

Our hypotheses were partially supported by the current data. Consistent with our first hypothesis, *T*_lim_ for exercise at 70, 80, and 90% 1RM were shorter than predicted from the severe domain relationship. Further, *Q*_tw_ and MVC were significantly decreased following severe intensity exercise and 60% 1RM, however, no change was detected following exercise at 70–90% 1RM by the time the reported measurements were made (~90 sec), while %VA was not significantly different pre‐to post‐exercise following any intensity. Furthermore, we found that EMG reached similar maximal (RMS) or minimum (MedPF) values across intensities above CP, independent of intensity. These EMG characteristics suggest maximal voluntary recruitment patterns at task failure, independent of exercise intensity for knee extension exercise. Most surprising, post hoc analysis showed exercise tolerance in the extreme domain elicited a hyperbolic power‐duration, giving evidence of a $W_{\mathrm{ext}}^{\prime }$.

### *T*_lim_ prediction

Time to task failure as a function of power in the severe intensity domain is hyperbolic versus time or linear as a function of 1/Time (Tornvall 1963; Monod and Scherrer 1965; Moritani et al. 1981; Poole et al. 1988; Vanhatalo et al. 2010; Broxterman et al. 2014). One interpretation could be that the mechanism(s) of fatigue limiting exercise tolerance in this domain are the same, independent of power. CP is thought to represent the highest level of sustainable aerobic metabolism (Poole et al. 1988; Burnley et al. 2012), and is significantly related to % type I fibers, and inversely related to % type IIx fibers of the contracting muscles (Vanhatalo et al. 2016). In contrast, *W*’ reflects, at least in part, anaerobic energy stores such as glycogen and PCr (Miura et al. 1999, 2000), but is not related to % type II fibers (Vanhatalo et al. 2016). During constant power intermittent contractions above CP, *T*_lim_ occurs when MVC falls below the target force requirement (Bigland‐Ritchie et al. 1986; Burnley et al. 2012). At task failure across varying intensities in the severe intensity domain, PCr, Pi, and H^+^ reach similar values (Jones et al. 2008; Vanhatalo et al. 2016), suggesting that *W*’ is dependent at least in part on anaerobic energy stores and/or the balance of appearance versus disappearance of fatigue inducing metabolites. Recent work has also demonstrated a link between the growing inefficiency of exercise in the severe (heavy) domain represented by the slow component of $\dot{V}$O_2_, which reflects the increased ATP cost of force production, and both *W*’ and fatigue (Grassi et al. 2015; Vanhatalo et al. 2016). In turn, this increased ATP cost of force production might reflect less free energy of ATP hydrolysis (less negative ΔG) at high metabolic rates (Grassi et al. 2015).

However, our data suggest these mechanisms are not (solely) responsible for task failure at all intensities above CP. If the process(es) producing fatigue were simply the same above as below S3, we would have predicted that *T*_lim_ for 70–90% 1RM would have fallen on the severe domain regression line. In contrast, at ~60% 1RM, we found *T*_lim_ were shorter than would be predicted (Figures 1 and 2). At intensities 70–90% 1RM, a separate linear model was necessary to describe the 1/Time relationship, with a greatly reduced slope compared to that of the severe domain. Because of the strong linear relationship, one interpretation may be that exercise tolerance in the extreme domain is limited or determined by mechanisms common within this domain, similar to the original interpretation of the hyperbolic nature of the severe domain. This demonstrates for the first time a unique work derivative for the extreme domain ($W_{\mathrm{ext}}^{\prime }$) which is smaller than *W*′. Hill et al. (2002) hypothesized that the upper threshold of the severe domain would occur at intensities that limit *T*_lim_ to less than 2 min. This hypothesis is consistent with our data for exercise following 70–90% 1RM, where task failure occurred on average within one minute and *T*_lim_ was much shorter than predicted, showing a different linear relationship above 60% 1RM. Thus, the region of the intersection of the two regression lines near 60% 1RM appears to reflect a phase transition from the severe to the extreme domain. Because the current protocol was not designed to carefully titrate a transition region between severe and extreme domains of exercise, we cannot say at this time if the intersection represents a true breakpoint response, or rather a region of power outputs for different subjects within which *T*_lim_ and the underlying mechanism(s) for task failure shift from one characteristic to another.

### Peripheral fatigue

Decreases in *Q*_tw_ have been used as evidence of peripheral fatigue (Bigland‐Ritchie et al. 1986). Following severe intensity exercise bouts to task failure, *Q*_tw_ is reduced to a similar degree across work rates (Burnley 2009; Burnley et al. 2012). Our data are consistent with these previous data; we show that decreases in *Q*_tw_ following exercise at S2–60% 1RM reached generally similar values. However, the decrease in *Q*_tw_ in the extreme domain (70%, 80%, and 90% 1RM) was not different among post‐exercise measurements. Further, *Q*_tw_ did not significantly decline from pre‐exercise baseline values following exercise at 80 and 90% 1RM. Broxterman et al. (2017b) observed a 52% reduction in *Q*_tw_ immediately following 5 min of an all‐out intermittent isometric exercise (60% MVC). It is currently unclear what the explanation is for this difference in response during extreme intensity exercise to task failure. Putative mechanisms could involve differences in contraction intensity (MVC vs. submaximal), duration (60 contractions vs. failure to produce target force), and time post task failure to muscle performance testing (immediate vs. 30 sec).

The finding that $W_{\mathrm{ext}}^{\prime }$ was significantly less than that of *W*′ suggests that the mechanisms responsible for task failure in the extreme domain (70–90% 1RM) may either be different from those in the severe intensity domain (S1–S3 and possibly 60% 1RM), or similar but with a lower threshold for exercise impairment. Within the severe domain, blood lactate, muscle Pi, and H^+^ continue to rise to, while PCr falls to, similar metabolite values until task failure across work rates (Vanhatalo et al. 2010; Jones et al. 2008; Vanhatalo et al. 2016; Black et al. 2017). Increases in both H^+^ and Pi have been shown to correlate with the decline in *Q*_tw_ during all‐out knee extension exercise (Blain et al. 2016). Our findings that *Q*_tw_ was significantly decreased following severe intensity exercise are consistent with these reported changes in muscle metabolites and their effects on muscle function. Within the extreme domain of exercise intensities, similar disturbances to cellular homeostasis have been observed. For example, following 1 bout of resistance exercise to task failure at 80% 1RM, PCr and muscle glycogen had fallen by 62% and 12%, respectively, while muscle lactate had increased by 12.5 fold (MacDougall et al. 1999). Given the similar reported metabolic changes with extreme exercise as with severe exercise, it is somewhat surprising that *Q*_tw_ was not significantly attenuated following exercise bouts at 80% and 90% 1RM; however, this could be due to methodological constraints. We are unaware of any study that reports metabolic responses to more than one exercise intensity in the same subjects in the extreme domain, so it is currently unclear if PCr, Pi, and pH reach similar common values across work rates in this domain, similar to what is observed for the severe domain. It is also currently unclear if these changes in PCr, Pi, and H^+^ in the extreme domain result in a similar reduction of the free energy of ATP hydrolysis (ΔG) at high metabolic rates (Grassi et al. 2015).

### Central fatigue

Consistent with our third hypothesis, the reduction in %VA following task failure was not statistically different for any of the exercise intensities above critical power. Increasing voluntary EMG, as seen in the present study by the attainment of maximal RMS at task failure for all exercise intensities, indicated increased excitatory input to the motoneuron pool, likely due to increased motor cortical input (Taylor et al. 2016). Given the relatively low % 1RM for CP in the current study (19% 1RM), our results are inconsistent with previous studies in elbow flexors demonstrating that central fatigue contributes significantly to exercise requiring lower force generation (≤20% MVC) (Smith et al. 2007; Yoon et al. 2007). However, our results at higher force production (≥30% MVC) are consistent with the observations that central fatigue was either modest (Bigland‐Ritchie et al. 1978; Yoon et al. 2007) or absent (Bigland‐Ritchie et al. 1986). Burnley et al. (2012) found that voluntary activation significantly declined for isometric knee extension tests to task failure at 38, 42, and 46% MVC suggesting central fatigue was present. However, at 50 and 55% MVC, there was no significant decline in %VA. This is consistent with the findings of Bigland‐Ritchie et al. (1986) for isometric knee extension exercise at 50% MVC. Similarly, Gruet et al. (2014) found that %VA had only dropped by 10% at task failure for intermittent contractions of 50% followed by 100% MVC. It is currently unclear where the variability in the presence and contribution of central fatigue as a function of force (as %MVC) originates from in previous studies, but could reflect differences in mode of exercise (cycling, knee extension, handgrip), contracting muscles (quadriceps, soleus, lower limb, forearm, biceps, triceps, first dorsal interosseous), and type of contraction (sustained isometric vs. intermittent, MVC vs. submaximal force), and interpretation of %VA should be taken with caution (de Haan et al. 2009).

Martin et al. (2006) found that certain central mechanisms may be restored relatively quickly. For example, cervicomedullary motor evoked potentials recovered within 15 sec following a 2 min MVC (Martin et al. 2006). This quick recovery of central mechanisms may shed light as to why exercise could not continue despite no recorded change in MVC or %VA following exercise at 80 and 90% 1RM. Several sites proximal to the neuromuscular junction that have been shown to contribute to fatigue in different conditions include fatigue of the motoneurons themselves (Johnson et al. 2004), a progressive decline in the discharge rate from muscle spindles (Macefield et al. 1991) associated with a decrease in the H‐reflex amplitude (Duchateau et al. 2002), and exercise‐induced changes in the neurotransmitters serotonin (5‐HT) and dopamine (Wei et al. 2014; Taylor et al. 2016). While their actions are complicated, muscle Golgi tendon organ output has been shown to have both inhibitory and facilitory influence on *α*‐motoneurons (for review see (Windhorst 2007)). Furthermore, it's possible the local environment created by intense force development during extreme intensity exercise stimulated a subpopulation of Group III/IV afferents sensitive to high‐metabolite concentrations (Amann et al. 2013; Blain et al. 2016), thus leading to reduced exercise tolerance.

### Experimental considerations

Several factors must be considered when interpreting the present data. First, the present study used a sample size of 6. This could be considered a relatively low sample size, however, our data show very consistent differences across the severe and extreme domains for all subjects. Our data also give evidence to a separate power/duration relationship of the extreme domain that reveals a much smaller *W*′ than that of the severe domain, and which was consistently observed in all subjects.

In order to enhance ecological validity and quantify any volitional changes in duty cycle from beginning to end exercise as well as differences from lighter to heavier loads, we did not control for duty cycle in the present study. Relaxation time was fixed at 1 sec in order to ensure constant time between contractions for blood flow (Broxterman et al. 2014). We found that within each subject, time of contraction (determined as length of EMG bursts) did not statistically change within individual exercise bouts or across exercise intensities. This suggests that individuals have an intrinsic contraction time that does not vary with heavier loads. However, we were unable to distinguish the concentric and eccentric portions of the contractions with EMG. Therefore, it may be possible that the concentric portion of the contraction gradually increased while the eccentric contraction was reduced. However, because total contraction times were constant and relatively short, we believe the any potential changes in concentric‐eccentric balance would have minimal effect.

Post‐exercise measurements in the current study were made as quickly as possible, and began 30 sec following exercise cessation. However, combined with the protocol used that discards the first two measurements, this may have been enough time for those central and peripheral factors that limited exercise to recover and therefore remain undetected (Desgorces et al. 2010; Froyd et al. 2013; Gruet et al. 2014). Irrespective of the underlying mechanism(s) for task failure in the 70–90% 1RM region, the hyperbolic nature of *T*_lim_ in this region implies a constant tolerance for exercise, analogous to the interpretation and findings associated with *W*’ for the severe domain (i.e., similar (in)tolerance to neural and intracellular metabolic perturbations across work rates).

## Conclusions

In conclusion, the current data demonstrated that the power‐duration relationship of the extreme intensity domain (70–90% 1RM) was hyperbolic, but not described by the power‐duration relationship for the severe intensity domain. Rather, above ~60% 1RM, a much smaller *W*′ ($W_{\mathrm{ext}}^{\prime }$) became evident which described the power‐duration relationship for 70–90% 1RM exercise. This hyperbolic power‐duration relationship implies a common mechanism for fatigue in the extreme domain (70–90% 1RM) that is different from those responsible for fatigue and task failure in the severe domain. Further, these mechanisms limiting extreme intensity exercise may be able to fully recover within 90 sec following exercise cessation, minimizing detection with the current protocol, and should be investigated in future studies.

## Conflict of Interest

The authors declare no competing interests.
